# Colocolonic intussusception of a colostomy after colonoscopy

**DOI:** 10.1093/jscr/rjac184

**Published:** 2022-05-25

**Authors:** Emmanuel Luciano, Omar Marar, Maxwell Cocco

**Affiliations:** Department of Surgery, Central Michigan University College of Medicine, Saginaw, MI, USA; Department of Surgery, Central Michigan University College of Medicine, Saginaw, MI, USA; Department of Surgery, Central Michigan University College of Medicine, Saginaw, MI, USA

## Abstract

The incidence of complications after colonoscopy is low and has been reported to range of 0.01–0.9%. Of these complications, colocolonic intussusception after colonoscopy is exceedingly rare, with around 12 known cases described in the literature. This case report details the presentation and operative management of a patient who developed an ischemic stoma due to a colocolonic intussusception of an end colostomy after a colonoscopy. Intraoperative surgical exploration revealed a colocolonic intussusception involving the end colostomy. This is the first known documented occurrence of this phenomenon.

## INTRODUCTION

Colonoscopy is a common diagnostic and therapeutic procedure with several associated risks, including perforation, hemorrhage and cardiac complications [1]. The incidence of complications is very low and has been reported to range from 0.01 to 0.9% [2]. Of these complications, colocolonic intussusception after colonoscopy is exceedingly rare, with around 12 known cases described in the literature [3]. Colonic intussusception due to any etiology is an exceptionally rare phenomenon in adults, with only 5% of cases occurring in this population [4]. Of the adult cases, 80–92% are due to a pathologic lead point and therefore the definitive management is resection [5, 6]. This case report details the operative management of colocolonic intussusception of a colostomy after a colonoscopy; a phenomenon that, based on a recent review of the literature, has never been described.

## CASE REPORT

**Figure 1:**
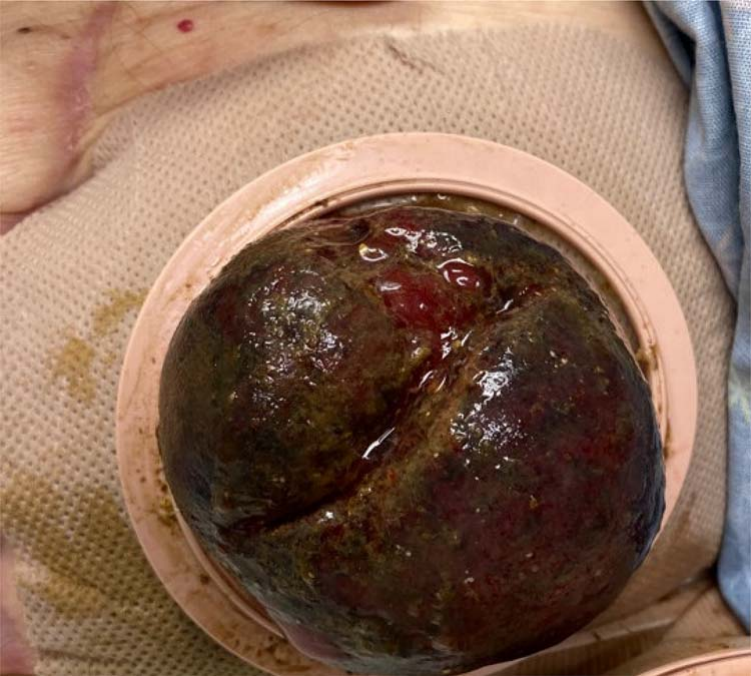
Edematous colostomy 7 days after colonoscopy.

**Figure 2:**
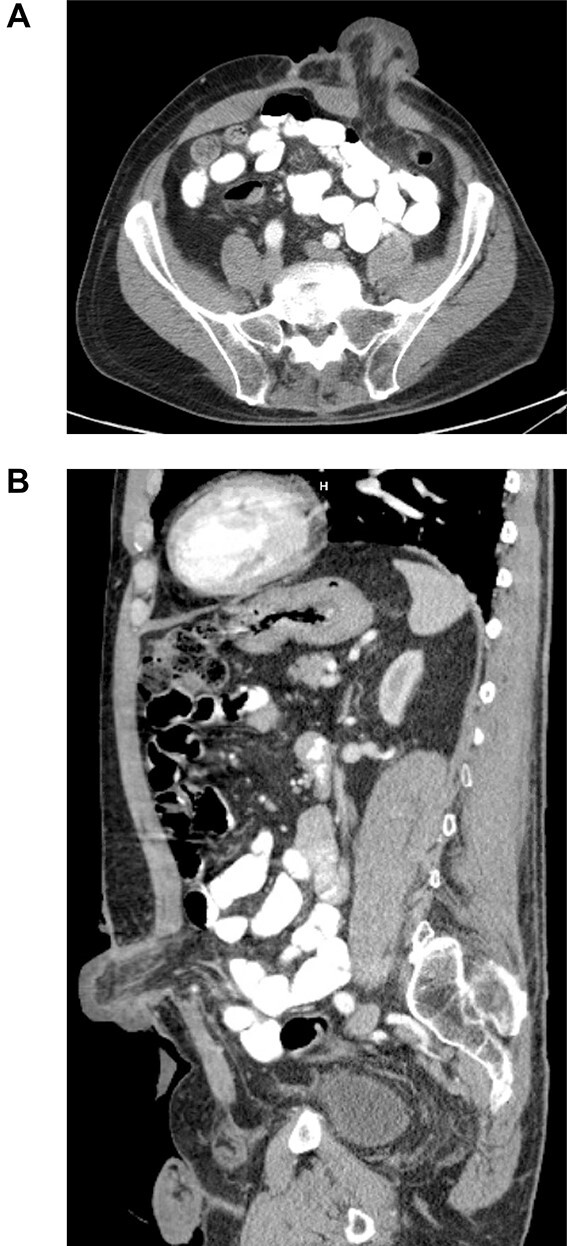
Axial (**A**) and sagittal (**B**) CT of the abdomen and pelvis, with IV contrast demonstrating edematous colostomy above the level of the fascia.

The patient is a 72-year-old male with a significant surgical history of an exploratory laparotomy with rectosigmoidectomy and end colostomy for a perforated stercoral ulcer 4 months prior to presentation. The patient was evaluated for colostomy reversal and underwent colonoscopic evaluation to assess the rectal stump and the remaining colon. The 10-cm rectal stump was found to have no abnormalities. The left lower quadrant colostomy was intubated, and the scope was advanced to the cecum and then carefully withdraw with circumferential evaluation of the mucosa. No polyps or abnormalities were identified. The patient tolerated the procedure well without any immediate complications and was discharged with planned colostomy reversal in the near future. The patient presented to the emergency department 1 week after the colonoscopy with a 1-day history of swelling and tenderness of the stoma with diminished output. On physical examination, there was no evidence of peritonitis, however, the stoma was edematous with evidence of ischemia (Fig. 1). Computed tomography (CT) of the abdomen and pelvis demonstrated edema of the stoma above the level of the fascia (Fig. 2). The patient was initially managed non-operatively without improvement and then the decision was made to take the patient to the operating room for colostomy reversal. A circumferential incision around the edematous stoma was made and this was dissected free from the subcutaneous and fascial attachments, which were minimal. The segment of stoma above the skin was ischemic, and upon mobilization of the colostomy, a colocolonic intussusception of the proximal colon into the colostomy was encountered. The intussusception was carefully reduced and the colon proximal to this area appeared to be well perfused, pink and viable. Following this, an exploratory laparotomy was performed and a standard stapled coloproctostomy was created. To protect the low anastomosis, a diverting loop ileostomy was created. The patient had an unremarkable post-operative course and was discharged from the hospital on post-operative day 3.

## DISCUSSION

Currently, there are several hypotheses that attempt to explain the phenomenon of intussusception after colonoscopy, including the theory of mucosal edema after polypectomy creating a lead point (proposed by Lee *et al*. in 2013) [7]. However, in this case, biopsies were not taken. Another theory proposed by Yamakazi et al. in 2000 suggests that a hyperperistaltic state induced by insufflation of the colon during colonoscopy could also induce intussusception [8]. This is more applicable to the complication observed in this case. Also contributing to the intussusception could be the vacuum effect induced by aspiration of gas on removal of the colonoscope, as described by Hassan and Teoh in 2018 [9].

Although both of these physiologic lead points are transient in nature, the presence of a stoma in this patient may have prevented an otherwise transient intussusception from resolving, resulting in the need for surgical intervention.

In summary, this is a unique case of colocolonic intussusception of a colostomy after a colonoscopy treated with reduction and colostomy reversal by stapled coloproctostomy. It is important to note that while the current definitive treatment for adult intussusception is operative management, cases of intussusception after colonoscopy have been reported to resolve, given the lack of a pathologic lead point [3]. However, in this case, the initial strategy of conservative management may have failed due to the unique nature and slightly different pathophysiology of the intussusception occurring involving the colostomy.
